# MicroRNAs are critical in regulating smooth muscle cell mineralization and apoptosis during vascular calcification

**DOI:** 10.1111/jcmm.16005

**Published:** 2020-10-22

**Authors:** Shan‐shan Wang, Chen Wang, Han Chen

**Affiliations:** ^1^ Department of Cardiology Zhejiang Provincial Key Lab of Cardiovascular Disease Diagnosis and Treatment Second Affiliated Hospital, Zhejiang University School of Medicine Hangzhou China

**Keywords:** apoptosis, microRNAs, smooth muscle cells, transdifferentiation, vascular calcification

## Abstract

Vascular calcification refers to the pathological deposition of calcium and phosphate minerals into the vasculature. It is prevalent in atherosclerosis, ageing, type 2 diabetes mellitus and chronic kidney disease, thus, increasing morbidity and mortality from these conditions. Vascular calcification shares similar mechanisms with bone mineralization, with smooth muscle cells playing a critical role in both processes. In the last decade, a variety of microRNAs have been identified as key regulators for the differentiation, phenotypic switch, proliferation, apoptosis, cytokine production and matrix deposition in vascular smooth muscle cells during vascular calcification. Therefore, this review mainly discusses the roles of microRNAs in the pathophysiological mechanisms of vascular calcification in smooth muscle cells and describes several interventions against vascular calcification by regulating microRNAs. As the exact mechanisms of calcification remain not fully elucidated, having a better understanding of microRNA involvement in vascular calcification may give impetus to development of novel therapeutics for the control and treatment of vascular calcification.

## 1 INTRODUCTION

Vascular calcification (VC) is the pathological deposition of calcium and phosphate minerals in the vasculature. It leads to vascular stiffness and fragility, impaired hemodynamics, and increased morbidity and mortality from cardiovascular diseases such as atherosclerosis, systolic hypertension and coronary artery disease_._ ^1^

Based on the location of hydroxyapatite precipitation, vascular calcification is classified into intimal and medial calcification.^2^ Intimal calcification is usually associated with atherosclerosis, in the presence of risk factors such as hyperlipidemia. Lipid deposited in the intima induces complicated pathophysiological responses, including inflammatory cell infiltration, endothelial cells (ECs) apoptosis, smooth muscle cells (SMCs) proliferation and transdifferentiation, extracellular matrix (ECM) remodelling and oxidative stress.^3^ Medial calcification is secondary to ageing, type 2 diabetes mellitus or chronic kidney disease (CKD), under the stimulation of hyperglycaemia and high circulating phosphate levels.^4^, ^5^ Epidemiological studies have highlighted that elevated inorganic phosphate (Pi) and calcium caused by disturbed mineral metabolism aggravates vascular calcification.^6^ Additionally, hyperglycaemia accelerates the accumulation of free radicals (superoxide anion) that can activate several cellular pathways including advanced glycation end products (AGEs), protein kinase C (PKC) and nuclear factor‐κB (NF‐κB)‐mediated vascular inflammation, which contribute to apatite formation in vasculature.^7^

Vascular smooth muscle cells (VSMCs) have been proven to play an essential role in both intimal and medial vascular calcification. This is characterized by VSMCs reprogramming and transdifferentiating into osteoblast‐like cells, VSMCs apoptosis and VSMCs‐derived calcifying matrix vesicle release. Besides VSMCs dysfunction, loss of calcification inhibitors, oxidative stress, endoplasmic reticulum stress and disturbed calcium‐phosphate homeostasis contribute to the development of calcification.^8^

MicroRNAs (miRs) are small non‐coding RNAs with 18‐25 nucleotides that bind to the 3’‐untranslated region of target messenger RNA (mRNA) to silence gene expression by destabilizing the mRNA or compromising mRNA translation. MicroRNAs regulate the expression of many genes and a multitude of cellular functions.^8^ In bone metabolism, miRs regulate the differentiation of bone precursor cells into mature bone cells. Likewise, a variety of miRs have been implicated in the development of vascular calcification. This review paper will introduce the role of miRs in the pathophysiological process of vascular calcification in VSMCs in order to identify potential therapeutics for vascular calcification associated diseases.

### 1.1 MicroRNAs in VSMCs osteochondrogenic transdifferentiation

During bone formation, bone marrow‐derived mesenchymal stem cells (MSCs) differentiate into chondrocytes or osteoblasts that are capable of synthesizing bone matrix and turning into osteocytes. Osteoblastic differentiation is regulated by hormones and various transcription factors. Bone morphogenetic proteins (BMPs), which are members of the transforming growth factor beta (TGF‐β) superfamily, mediate transdifferentiation of MSCs into osteoblasts through BMP/Smad signalling pathway.^9^ The BMPs were identified to target Runx2 and Osterix in the process of bone formation.^9^ As a member of the Runt‐related transcription factors, Runx2 is the master upstream osteoblast transcription factor that regulates opulent bone matrix proteins expression.^10^ In the transcriptional cascade of osteoblast differentiation, Msx2 and Osterix act as the upstream and downstream connectors of Runx2, respectively.^10^, ^11^ Moreover, BMPs can activate Wnt/β‐catenin signalling pathway to promote alkaline phosphatase (ALP) expression and matrix mineralization.^12^

Vascular calcification and bone mineralization share similar mechanisms.^2^ At the molecular level, the signature of active osteogenic processes is found in virtually all calcified arterial segments.^13^ The VSMCs normally express contractile molecules, including smooth muscle actin‐α (α‐SMA), transgelin (SM22a), smooth muscle myosin heavy chain (SM‐MHC) and calponin 1 (CNN1).^14^ However, when exposed to atherogenic or uraemic stimulus, they are capable of transdifferentiating into osteo/chondrocyte‐like cells. This induces increased expression of bone‐related transcription factors such as Msx2, Sox9, Runx2, Osterix, tissue non‐specific alkaline phosphatase (TNAP), osteocalcin and osteopontin (OPN).^9^, ^15^, ^16^

**Figure 1:**
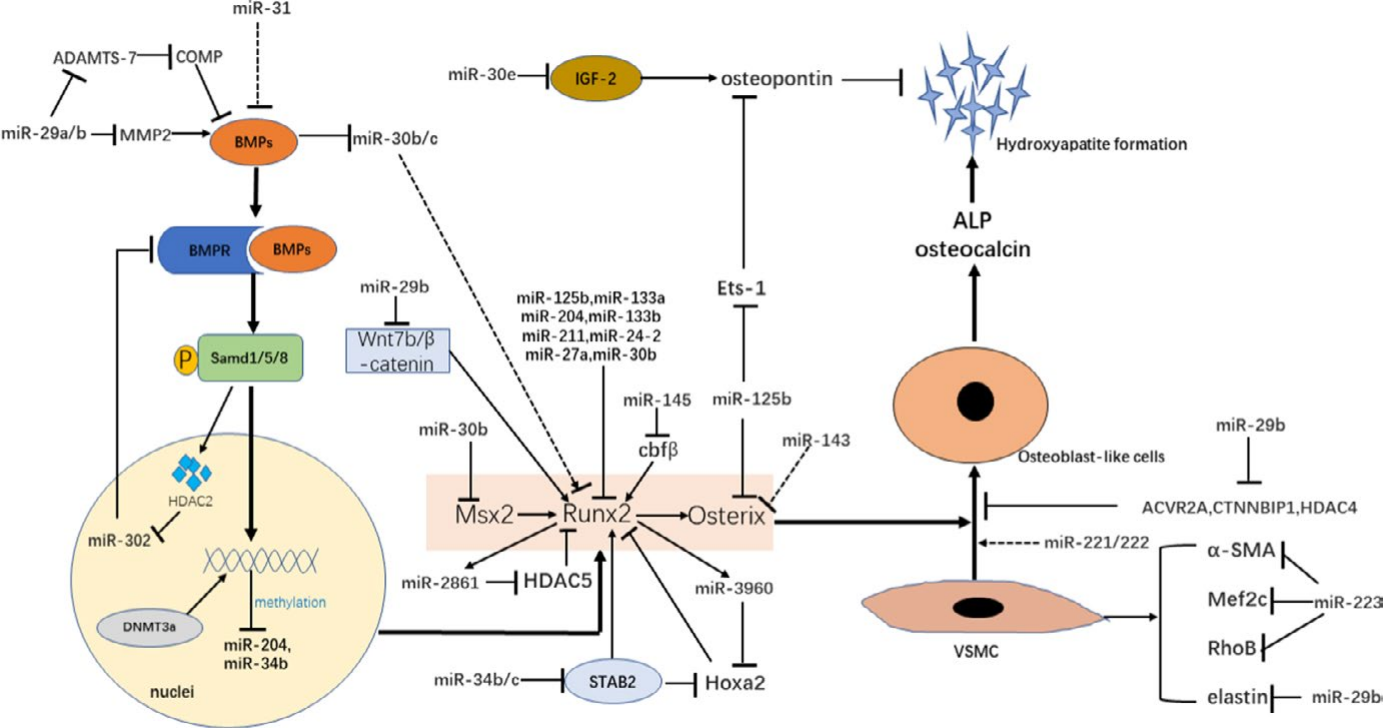
Schematic representation of microRNAs in osteogenic transdifferentiation of VSMCs. Upon BMPs binding to the receptor complex, Smad proteins translocate into the nucleus and modulate gene expression transcriptionally by directly interacting with the promoter region of target genes (such as Runx2 and Osterix) or post‐transcriptionally through regulating miRs synthesis. Then, these osteoblast transcription factors regulate opulent bone matrix proteins expression and promote transdifferentiation of VSMCs to osteoblast‐like cells. MicroRNAs regulate osteogenic transdifferentiation of VSMCs through targeting key factors in the BMP/Smad/Runx2 signalling pathway and several inhibitors of osteoblastic differentiation. Besides, some miRs are able to regulate the expression of proteins involved in VSMCs differentiation, migration and contractility. ADAMTS‐7, a disintegrin and metalloproteinase with thrombospondin motifs 7; ACVR2A, A receptor type IIA; ALP, alkaline phosphatase; BMPR, Bone morphogenetic proteins receptor; BMPs, Bone morphogenetic proteins; Cbfβ, core‐binding factor beta; COMP, cartilage oligomeric matrix protein; CTNNBIP1, b‐catenin interacting protein 1; DNMT3a, DNA methyltransferase 3A; Ets1, endothelial transcription factor 1; HDAC2, histone deacetylase 2; HDAC4, histone deacetylase 4; Hoxa2, Homeobox A2; IGF‐2, Insulin‐like growth factor 2; Mef2c, myocyte enhancer factor 2C; MMP2, matrix metallopeptidase 2; RhoB, ras homolog gene family member B; SATB2, Special AT‐rich sequence‐binding protein 2; Wnt, wingless‐type MMTV integration site; α‐SMA, smooth muscle actin‐α. 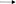 stimulation; 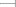 inhibition

Studies have demonstrated that MiRs are essential regulators for osteoblast transdifferentiation of VSMCs. The majority of reported miRs are down‐regulated during the process of SMCs transdifferentiation whereas some of them are up‐regulated in this process. The regulation of osteogenic transdifferentiation of VSMCs by miRs is illustrated in Figure 1.

Upon BMPs binding to the receptor complex, Smad proteins translocate into the nucleus and modulate gene expression transcriptionally by directly interacting with the promoter region of target genes or post‐transcriptionally through regulating miRs synthesis. The BMPs suppress miR‐302 transcription via a Smad protein complex in a histone deacetylase (HDAC)‐dependent manner. This results in elevation in the expression of BMP receptor II, which consequently improves BMP signalling in VSMCs.^17^ Sun et al^18^ found that overexpression of miR‐302b in mice with chronic renal failure regulated calcium‐phosphorus metabolism and inhibited VC through down‐regulating BMP receptor II expression, which inhibits BMP‐2/Runx2/Osterix pathway.

In another study, miR‐125b was reported to regulate cell proliferation during osteoblast differentiation in mouse MSCs.^19^ Additionally, Goettsch et al^20^ uncovered that miR‐125b expression was significantly reduced in calcifying human coronary artery smooth muscle cells (HCASMCs) cultured in an osteogenic medium and in calcified aortas of apolipoprotein E (ApoE) deficient mice. However, mRNA expression of Runx2 and ALP was increased in these cells. ALP promotes vascular calcification by reducing the production of inorganic pyrophosphate, which is a potent inhibitor of VC.^21^ Similarly, Wen et al^22^ reported down‐regulation of expression of miR‐125b in rat aortic VSMCs calcification induced by β‐glycerophosphate (β‐GP). Knock‐down of miR‐125b aggravated osteogenic transdifferentiation and calcium deposition in VSMCs via increasing Osterix and Ets‐1 protein expression.^20^, ^22^ The Ets1 is a potent transcription factor that promotes VSMCs remodelling through regulating genes encoding extracellular matrix proteins, such as osteopontin.^23^

Rangrez and colleagues^24^ found that miR‐143 and miR‐145 were down‐regulated whereas miR‐223 was markedly up‐regulated in calcified VSMCs treated with high Pi and calcified aorta samples derived from ApoE deficient mice. However, circulating levels of miR‐223 were significantly lower in CKD mice and in a cohort of patients with advanced CKD, while the expression of miR‐223 increased in the calcified vascular SMCs, which indicated an accumulation of this microRNA in the vascular wall.^25^, ^26^ In VSMCs that were treated with high Pi, overexpression of miR‐223 markedly inhibited α‐SMA, improved VSMCs migration and induced calcification by targeting myocyte enhancer factor 2C (Mef2c) and ras homolog gene family member B (RhoB).^24^ The Mef2c is involved in VSMCs differentiation and regulates myocardin(MYOCD) expression^27^ whereas RhoB is a member of the Rho guanosine triphosphatases family of proteins and has been shown to increase VSMCs contractility and mediate adaptational changes to hypoxia.^28^ Another microRNA, MiR‐143, suppresses osteogenic differentiation via targeting transcription factor Osterix and is potentially related to VC^29^ whereas MiR‐145 was reported to target core‐binding factor‐beta (Cbfβ), which is the hetero‐dimeric partner of Runx2, to transactivate molecular target of Runx2 in osteoblast differentiation.^30^

There are several miRs that have been demonstrated to regulate VSMCs transdifferentiation during VC via targeting the osteoblastic marker Runx2. When treated with β‐GP, VSMCs showed increased Runx2 expression along with increased calcium deposition, while expression of miR‐133a and miR‐204 were significantly inhibited.^31^, ^32^ Notably, Lin et al^33^ elucidated that down‐regulation of miR‐204 in calcified arteries was associated with DNA methylation induced by DNA methyltransferase 3A (DNMT3a). Knock‐down of DNMT3a restored the expression of miR‐204 and successfully suppressed the expression of osteoblastic markers expression induced by high‐phosphate medium in the human aortic vascular smooth muscle cell line (HAVSMCs). Similarly, down‐regulation of miR‐133b and miR‐211 was suggested to target osteoblastic marker Runx2 in VC processes in uraemic mouse and high Pi‐cultured murine VSMCs models.^34^

Down‐regulation of miR‐221 induces osteogenic differentiation in human MSCs, highlighting the potential involvement of this microRNA in VC.^35^ A study by Mackenzie et al^36^ demonstrated that miR‐221, miR‐222, miR‐24‐2, miR‐27a and miR‐31 were significantly down‐regulated in cultured murine VSMCs treated with phosphate‐rich medium. Nonetheless, transfected VSMCs with mimics of miR‐221 and miR‐222 were reported to synergistically improve the transdifferentiation of VSMCs and calcium deposition in vitro, independent of Runx2 and Msx2 expression. Mackenzie et al^36^ speculated miR‐221 and miR‐222 mainly exert a mediating role in the initial stage of cellular transdifferentiation and then degenerated when cells progress towards an osteochondrogenic phenotype. Besides, Chistiakov et al^37^ speculated that chronic inflammation within vasculature promotes osteogenic/chondrogenic phenotype transition of VSMCs partly through inhibiting miR‐221/222 expression and then induces neointimal formation, intimal thickening and ectopic calcification of atherosclerotic arteries. Additionally, miR‐31 was reported to suppress TNAP activity in differentiating human bone marrow multipotent MSCs via regulating Runx2 and BMP2 expression, suggesting down‐regulation of miR‐31 in calcified vessels may similarly regulate osteo‐transdifferentiation of VSMCs.^38^

Members of the miR‐30 family were reported to suppress osteoblast differentiation in mouse bone marrow MSCs induced by BMP‐2 through targeting Smad1 and Runx2.^39^ For instance, MiR‐30e is reciprocally associated with osteoblast and adipocyte differentiation.^40^ Overexpression of miR‐30e improved adipogenesis but repressed osteoblast differentiation in mouse bone marrow stromal cells by down‐regulating Wnt/β‐catenin signalling.^40^ Additionally, significantly reduced expression of miR‐30e and up‐regulated Runx2, OPN and insulin‐like growth factor 2 (IGF‐2) were reported in aortas from aged ApoE deficient mice. As one of the targets of miR‐30e, IGF‐2 promotes OPN expression and calcium deposition.^41^ It is probable that BMPs mediates phenotype switch of VSMCs during VC by regulating the expression of miRs. However, miRs are also capable of influencing BMPs‐induced osteoblast differentiation by targeting osteoblastic transcription factors. Balderman et al^42^ proved that BMP‐2 markedly improved Runx2 protein expression by decreasing miR‐30b and miR‐30c expression in calcified HCASMCs, whereas another study reported that miR‐30b inhibited BMP2‐induced osteoblast differentiation via down‐regulating Runx2 and Msx2 gene expression.^43^

In BMP2‐induced murine bone osteoblast differentiation, the increased expression of miR‐34 significantly suppressed osteoblasts proliferation by targeting the Notch signalling pathway to inhibit Cyclin D1, CDK4 and CDK6 accumulation. It also reduces osteoblasts terminal differentiation through repressing special AT‐rich sequence‐binding protein 2 (SATB2) and increased osteoclastogenesis.^44^, ^45^ SATB2 positively regulates osteoblast differentiation via promoting Runx2 transcription during bone formation.^46^ Hao et al^47^ identified that miR‐34b/c expression was significantly decreased whereas SATB2/Runx2 protein, ALP activity, osteocalcin secretion and calcium content were up‐regulated in VSMCs treated with aldosterone. Furthermore, they demonstrated that overexpression of miR‐34b/c attenuates aldosterone‐induced VSMCs calcification via SATB2/Runx2 pathway. Similar to miR‐204, down‐regulation of miR‐34b in calcified aortas was also consequence of upstream DNA methylation regulated by DNMT3a, and restoring miR‐34b expression attenuated vascular calcification in vitro through Notch1.^48^

Several studies showed that miR‐29a/b expression was markedly decreased while a disintegrin and metalloproteinase with thrombospondin motifs 7 (ADAMTS‐7) and matrix metallopeptidase 2 (MMP2) were significantly increased in rat VC models and VSMCs induced by β‐GP, high calcium or cholecalciferol.^49^, ^50^, ^51^ These two genes, ADAMTS‐7 and MMP2, are members of the metalloproteinase family. The former mediates degradation of cartilage oligomeric matrix protein (COMP) in injured vessels.^52^, ^53^ COMP inhibits VSMCs calcification via suppressing BMP2 expression and inhibiting osteo/chondrogenic transdifferentiation. Hence, studies have reported down‐regulated COMP in calcifying arteries.^54^ Down‐regulation of miR‐29a/b led to ADAMTS‐7 overexpression, excess COMP degradation, enhanced BMP‐2 osteogenic signalling and aggravated calcium deposition in high‐phosphate‐induced human VSMCs and calcium chloride (CaCl_2_) induced rat VSMCs.^51^ MMP2 induces osteoblastic transdifferentiation of VSMCs via increasing BMP‐2 expression, and subsequently increasing the expression of Runx2 and Msx‐2.^55^ However, overexpression of miR‐29b‐3p was reported to repress MMP2 expression and then ameliorate VSMC calcification.^49^, ^50^ Besides, in human aortic smooth muscle cells (HASMCs) calcification model, miR‐29b mimics were demonstrated to markedly attenuate Wnt7b/β‐catenin protein overexpression, suppress Runx2 osteogenic signalling and osteopontin (OPN) expression.^56^ Nonetheless, another two studies reported notable up‐regulation of miR‐29b in the process of osteoblastic transdifferentiation in uraemic mouse and high Pi‐treated VSMCs models. Overexpression of miR‐29b was found to reduce the expression of elastin mRNA^57^ and several inhibitors of osteoblastic differentiation including activin A receptor type IIA (ACVR2A), β‐catenin interacting protein 1 (CTNNBIP1) and histone deacetylase 4 (HDAC4).^31^ Added together, the differences in the expression patterns of miR‐29 reported in different studies imply that miR‐29 might play essential role in Pi‐mediated calcification.

The Runx2/miR‐3960/miR‐2861 positive feedback loop also contributes to osteoblast differentiation. During the process of osteoblast differentiation, Runx2 transactivates miR‐3960/miR‐2861 and induces genes requisite for osteoblast differentiation. In turn, miR‐3960 targets Homeobox A2 (Hoxa2) and miR‐2861 targets histone deacetylase 5 (HDAC5) to increase Runx2 expression and acetylation, resulting in improvement of osteoblast differentiation.^58^ In conformity with that of bone mineralization, it is reported that up‐regulation of miR‐2861 and miR‐3960 in β‐GP induced mice VSMCs improve the expression of osteoblastic markers through decreasing HDAC5 and Hoxa2 expression. Additionally, Runx2 activation could directly enhance the expression of miR‐2861 and miR‐3960 in VSMCs.^59^

### 1.2 MicroRNAs in VSMCs apoptosis

**Figure 2:**
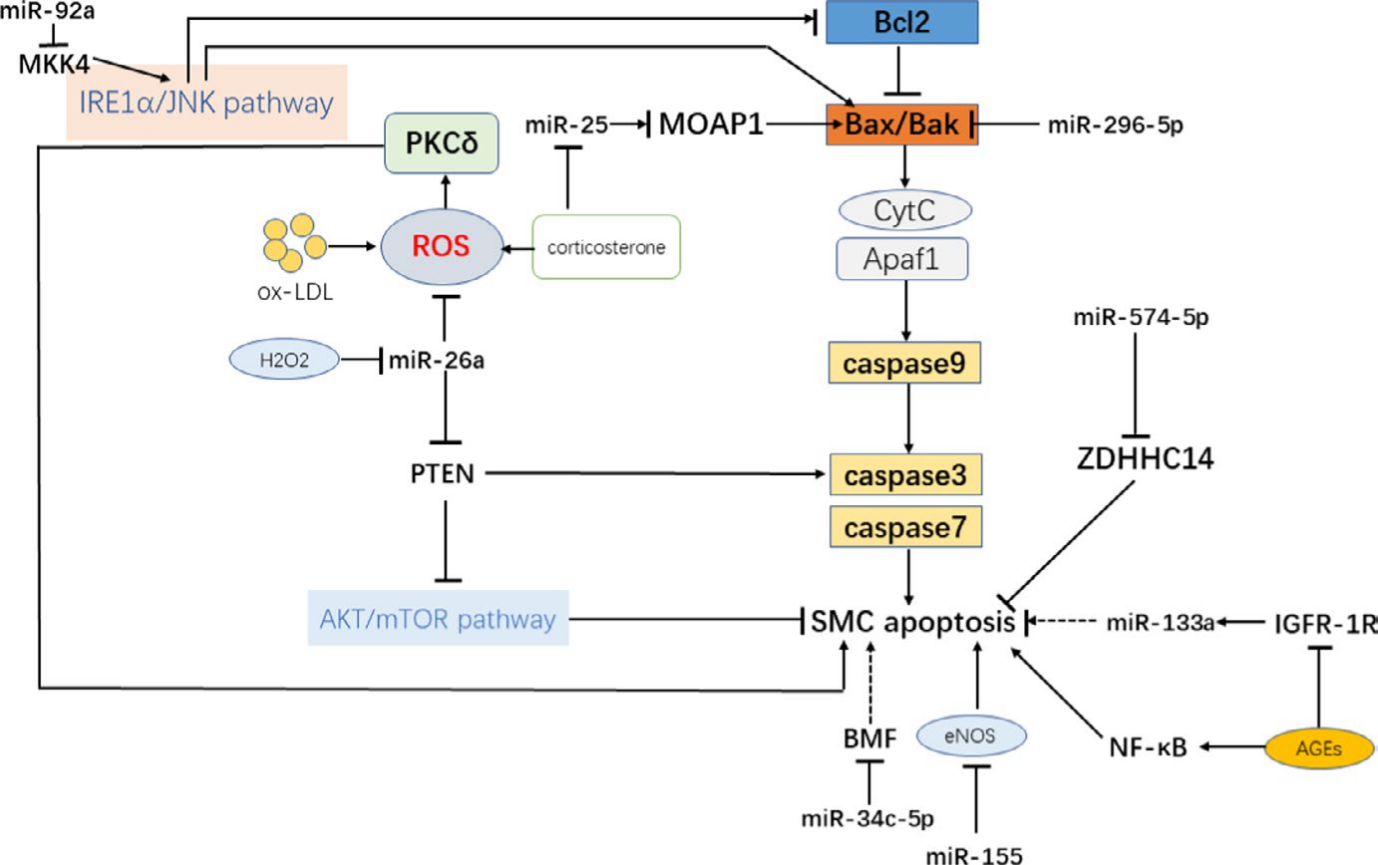
Overview of microRNAs in apoptosis of VSMCs. The intrinsic cellular apoptosis pathway is initiated by caspases activation and mitochondrial changes, which are regulated by Bcl‐2 family members. MicroRNAs regulate apoptosis of VSMCs via targeting anti‐/pro‐apoptotic proteins in the cellular apoptosis pathway or regulating factors of apoptosis. AGEs, advanced glycation end products; BMF, Bcl‐2 modifying factor; eNOS, endothelial nitric oxide synthase; IGFR‐1R, insulin growth factor 1 receptors; MKK4, Mitogen‐activated protein kinase kinase 4; MOAP1, Modulator of apoptosis 1; mTOR, mammalian target of rapamycin; PKCδ, protein kinase Cδ; PTEN, phosphatase and tensin homologs. 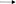 stimulation; 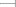 inhibition

Apoptosis of VSMCs is a key process in vascular calcification. The intrinsic cellular apoptosis pathway is initiated by caspases activation and mitochondrial changes, which are regulated by Bcl‐2 family members.^60^ These Bcl‐2 family members include anti‐apoptotic proteins such as Bcl‐2, Bcl‐x, Bcl‐XL, Bcl‐XS, Bcl‐w, BAG and pro‐apoptotic proteins such as Bcl‐10, Bax, Bak, Bid, Bad, Bim, Bik and Blk. Matrix vesicles are regarded as the nucleation sites for calcium crystal formation in bone. Apoptotic bodies derived from VSMC act similarly as nucleating structures for calcium crystal formation during vascular calcification.^61^ Series of studies have reported that miRs are pivotal regulators of VSMCs apoptosis in the process of vascular calcification. The process through which miRs regulate apoptosis of VSMCs is shown in Figure 2.

Several miRs have been identified to positively regulate VSMCs apoptosis. Overexpression of miR‐34 family members was reported to promote apoptosis of VSMCs and Umbilical Vein Endothelial Cells (UVECs) in a caspase‐3‐dependent manner.^62^ Choe et al^63^ demonstrated that transfection of miR‐34c to VSMCs induced apoptosis accompanied by up‐regulation of p21, p27 and Bax in an animal model. Another study using high Pi‐cultured mice SMCs and proliferative/young HASMCs found that miR‐34a overexpression induced VSMCs apoptosis and promoted calcium deposition through negatively regulating the expression of longevity‐associated gene sirtuin 1 (SIRT1) and anti‐apoptotic receptor tyrosine kinase Axl.^64^ Zong et al^65^ reported that overexpression of miR‐30e promoted VSMCs apoptosis and suppressed their proliferation and migration in vitro. Furthermore, miR‐132 induced VSMCs apoptosis and blocked proliferation through leucine‐rich repeat (in Flightless 1) interacting protein‐1 (LRRFIP1).^66^ It is also noteworthy that miR‐29b positively modulates apoptosis of VSMCS in primary cultured HASMCs through promoting caspase‐3 activity.^67^ In addition, Chen et al^68^ revealed that exogenous miR‐125b promoted VSMCs apoptosis and inhibited cell proliferation and migration in arterial specimen medium via targeting serum response factor (SRF).

On the contrary, some miRs prevent apoptosis of VSMCs, thus possibly play a role in ameliorating vascular calcification.

A few miRs are capable of suppressing oxidative stress‐induced apoptosis. During atherogenesis, reactive oxygen species (ROS) generated by oxidized LDL (oxLDL) activate protein kinase Cδ (PKCδ), which regulates oxLDL‐induced endoplasmic reticulum (ER) stress‐mediated apoptosis in VSMCs, mainly via the IRE1α/JNK pathway.^69^ Mitogen‐activated protein kinase kinase 4 (MKK4) was found to directly phosphorylate and activate JNK in response to environmental stress and pro‐inflammatory cytokines.^70^ A study reported that expression of miR‐92a was suppressed by H_2_O_2_‐induced oxidative stress and that overexpressing miR‐92a protected VSMCs against apoptosis from oxidative stress partly via down‐regulating MKK4/JNK1 signalling pathway.^71^ Similarly, Peng et al^72^ demonstrated that decreased expression of miR‐26a was associated with severe apoptosis and increased ROS production in H_2_O_2_‐treated VSMCs. Up‐regulation of miR‐26a attenuated VSMCs apoptosis via targeting phosphatase and tensin homologs (PTEN), which significantly induced apoptosis through promoting cleavage of caspase‐3 in primary rabbit VSMCs.^73^ Nevertheless, Lin et al^74^ reported that high H_2_O_2_ accelerated VSMC apoptosis and up‐regulated the expression of miR‐21. Overexpression of miR‐21 was demonstrated to suppress H_2_O_2_‐regulated VSMCs apoptosis via pro‐apoptotic protein PDCD4.

Zhang et al^75^ indicated that corticosterone also induced ROS production and apoptosis of VSMCs, coincident with significantly decreased expression of miR‐25. Notably, the up‐regulation of miR‐25 attenuates corticosterone‐induced apoptosis in VSMCs by targeting pro‐apoptotic protein Modulator of apoptosis 1 (MOAP1) and inhibiting p70S6K activity.^75^ MOAP1 induces caspase‐dependent apoptosis in mammalian cells via binding pro‐apoptotic BAX through its Bcl‐2 homology‐3‐ like motif.^76^

Hyperglycaemia and advanced glycation end products (AGEs) were reported to down‐regulate the expression of miR‐133a and then aggravate apoptotic susceptibility of VSMCs via inhibiting insulin growth factor 1 receptor (IGFR‐1R) activity and activation of NF‐κB. This indicated miR‐133a may protect against VSMCs apoptosis in diabetes mellitus.^77^ In addition, diabetic serum‐derived‐extracellular vesicles (DCD31EVs) enriched with platelet‐derived growth factor B‐chain (PDGF‐BB) were shown to increase miR‐296‐5p level, which down‐regulates apoptosis of VSMCs through diminishing the expression of pro‐apoptotic protein Bak.^78^

Another gene, MiR‐34c‐5p, was verified to be directly inhibited by lncRNA‐ES3 (an endogenous RNA that competes with miR‐34c‐5p) to aggravate high glucose‐induced HAVSMCs calcification via promoting its target Bcl‐2 modifying factor (BMF) expression.^79^ There was up‐regulation of BMF in mice renal proximal tubular cells (RPTCs) treated with high glucose to promote apoptosis of RPTCs.^80^ This suggested that BMF might similarly mediate apoptosis of VSMCs during vascular calcification targeted by miR‐34c‐5p. Exogenous overexpression of miR‐155 notably improved SMCs proliferation via reducing apoptosis in HASMCs through targeting endothelial nitric oxide synthase (eNOS).^81^ Another study showed that increased expression miR‐574‐5p in the sera and VSMCs of patients with coronary artery disease suppressed apoptosis of VSMCs but improved VSMCs proliferation.^82^

### 1.3 MicroRNAs in the treatment of vascular calcification

In recent years, in‐depth studies on the pathophysiological mechanism of vascular calcification have reported several interventions to inhibit VC through regulating miRs expression.

Melatonin (MT) was shown to exert a biological role of clearing free radicals, antioxidant, anti‐inflammatory and playing epigenetic regulatory functions.^83^ Xu et al^84^ exhibited that exosomes secreted by melatonin‐treated VSMCs repressed osteogenic differentiation and reduced vascular calcification through a paracrine effect of exosomal miR‐204/miR‐211. In another study, Guo et al^85^ confirmed that bone marrow mesenchymal stem cell (BMSC)‐derived exosomes decreased calcium content and inhibited ALP activity in high phosphorus induced calcification of HASMCs via regulating mRNA and microRNA expression profiles. They speculated that miRs in BMSC‐derived exosomes modulate wingless‐type MMTV integration site (Wnt), mammalian target of rapamycin (mTOR) and mitogen‐activated protein kinase (MAPK) signalling pathways, which are indispensable to the process of vascular calcification.

Topoisomerase II inhibitor (Topo II inhibitor) was reported to impair macrophage and SMCs invasion of the intima and inhibit the expression of inflammatory cytokines in the arterial wall.^86^ Liu et al^87^ demonstrated that teniposide, one type of DNA Topo II inhibitors, inhibited BMP2/p‐Smad1/5/8/Runx2 signalling pathway and decreased vascular calcium accumulation in ApoE deficient mice and HASMCs via up‐regulating miR‐203‐3p expression. In addition, Han et al^88^ found that when HASMCs were treated with teniposide there was enhanced expression of SMA and SM22α. However, they reported reduced osteopontin expression via promoting the expression of miR‐21. Notably, SRF is the main transcription factor controlling SMC phenotype switching, which can be activated by MYOCD.^89^ Teniposide up‐regulates expression of SRF and MYOCD while suppressing Msx1 and Msx2 expression, suggesting teniposide may inhibit SMC phenotype switch during VC through improving contractile genes expression and reducing Msx1/2 expression by activation of SRF‐MYOCD activity and increase of miR‐21 expression.^88^

Iron, which is capable of decreasing phosphate load and regulating phosphate exosome content, is used to treat hyperphosphatemia in CKD patients as the Pi‐binder.^90^ A study found that high‐phosphate‐induced vascular calcification was suppressed by iron citrate via reducing phosphate load and up‐regulating the expression of miR‐30c. Researchers also demonstrated that iron both prevented and partially reversed collagen deposition and the elastin decrease in high‐phosphate cultured rat aortic tissue.^91^

As shown before, patients with CKD are more susceptible to VC. Claudia et al^92^ discovered that plasma extracellular vesicles (EV) from bicarbonate hemodialysis (BHD) patients increased the expression of proatherogenic miR‐223 and induced VSMC calcification when compared with that from healthy individuals. However, mixed online hemodiafiltration (mOL‐HDF) treatment could reduce VSMC calcification via inhibiting the expression of miR‐223 in plasma EV.^92^ This result indicates that mOL‐HDF treatment may be beneficial for reducing the morbidity of cardiovascular events among CKD patients.

Wang et al showed that Poly (ADP‐ribose) polymerase 1 (PARP1) was up‐regulated in radial artery samples from patients with chronic renal failure, in arteries from uraemic rats and in calcified VSMCs in vitro. Overexpression of PARP1 increased the expression of Runx2 and exacerbated calcium deposition through suppressing miR‐204 expression via the IL‐6/STAT3 pathway.^93^ As a result, therapeutic agents that regulate PARP1 activity may be used to relieve vascular calcification.

## 2 CONCLUSION

In this review, we mainly discussed the involvement of miRs in osteogenic transdifferentiation and apoptosis of SMCs during the process of VC. It was found that numerous miRs regulate hydroxyapatite formation in the vasculature through targeting specific genes. In addition, the BMP/Smad/Runx2 signalling pathway is considered as the foremost pathway during VC. Therefore, the miRs regulating this pathway contribute to the process of VC. Some miRs are modulated through several interventions, which may be used in development of potential therapeutics in the future to control VC and prevent the development of cardiovascular diseases. Apart from playing a role in the reprogramming and transdifferentiating of SMCs, studies demonstrated that miRs are also associated with SMCs autophagy, increased SMCs oxidants and/or endoplasmic reticulum stress and loss of calcium‐phosphate homeostasis.^94^, ^95^, ^96^ To summarize, miRs involvement in vascular calcification is pretty complicated; hence, more studies are needed to fully explore the association between miRs and VC and discover more targets for VC therapeutic intervention.

## CONFLICT OF INTEREST

The authors declare that they have no competing interests.

## AUTHOR CONTRIBUTION

**Shan‐shan Wang:** Conceptualization (equal); Investigation (equal); Writing‐review & editing (equal). **Chen Wang:** Funding acquisition (supporting); Visualization (equal). **Han Chen:** Conceptualization (lead); Resources (lead); Supervision (lead); Writing‐review & editing (lead).
